# Flip Chip Bonding of a Quartz MEMS-Based Vibrating Beam Accelerometer

**DOI:** 10.3390/s150922049

**Published:** 2015-09-02

**Authors:** Jinxing Liang, Liyuan Zhang, Ling Wang, Yuan Dong, Toshitsugu Ueda

**Affiliations:** 1 Key Laboratory of Micro-Inertial Instrument and Advanced Navigation Technology, Ministry of Education, School of Instrument Science and Engineering, Southeast University, Nanjing 210096, China; E-Mails: violetyuan7@126.com (L.Z.); 213113391@seu.edu.cn (Y.D.); 2 School of Automation, Southeast University, Nanjing 210096, China; E-Mail: 213122024@seu.edu.cn; 3 Graduate School of Information, Production and Systems, Waseda University, Kitakyushu 808-0135, Japan; E-Mail: t-ueda@waseda.jp

**Keywords:** quartz MEMS, vibrating beam accelerometer, flip chip bonding, self-alignment, double ended tuning fork

## Abstract

In this study, a novel method to assemble a micro-accelerometer by a flip chip bonding technique is proposed and demonstrated. Both the main two parts of the accelerometer, a double-ended tuning fork and a base-proof mass structure, are fabricated using a quartz wet etching process on Z cut quartz wafers with a thickness of 100 μm and 300 μm, respectively. The finite element method is used to simulate the vibration mode and optimize the sensing element structure. Taking advantage of self-alignment function of the flip chip bonding process, the two parts were precisely bonded at the desired joint position via AuSn solder. Experimental demonstrations were performed on a maximum scale of 4 × 8 mm^2^ chip, and high sensitivity up to 9.55 Hz/g with a DETF resonator and a Q value of 5000 in air was achieved.

## 1. Introduction

**Figure 1:**
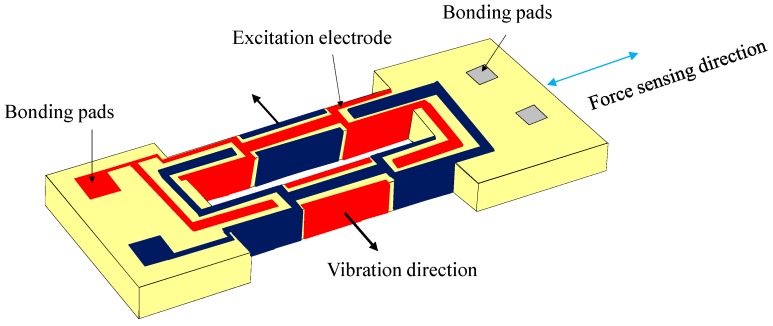
Schematic diagram of double-ended tuning fork and excitation electrode configuration.

Recently resonator-based micro-accelerometers have drawn great attention due to their digital frequency output without the need of analog/digital conversion, which may induce additional errors [1,2,3,4,5]. Although most micro-resonant accelerometers are fabricated on silicon wafers, quartz-based vibrating beam accelerometers (QVBAs) have also been studied for a long time owing to their simple structure, high frequency stability and so on [6]. The key force sensing element vibrates in an in-plane flexural mode, which could be a single-beam or a double-beam resonator, also called a double-ended tuning fork (DETF) [7] The single-beam-based QVBA has twice the sensitivity of a double-beam one for the same converted inertial force, however a complex vibration isolation structure is needed to reduce the vibrational energy leaking into the mounting parts [8,9]. The DETF-based QVBA is constructed from a double-ended tuning fork (DETF) force-frequency resonator and a proof mass structure [10]. The DETF works in an anti-phase in-plane flexural mode and the two beams vibrate in the opposite direction, canceling the force and torque produced at the two joint roots. Taking advantage of the piezoelectric effect of quartz material, the flexural vibration can be easily excited by depositing metal film electrodes on the beams with corresponding electrode pattern as shown in Figure 1 [11]. The proof mass structure is composed of a base and a proof mass, which are connected by a thinned flexure. The two ends of the quartz DETF are jointed on the base and proof mass surface, respectively. 

This kind of sensor measures the acceleration information in a direction perpendicular to the proof mass surface, which is converted into so called inertial force. The induced inertial force causes the proof mass to rotate slightly around the fixed base mainly by the deformation of the thinned flexure, which produces a longitudinal force on the DETF resonator. The resonating frequency of the DETF could increase upon a tension or decrease upon a compression. Currently, the proof mass structure is usually made by steel material to achieve a relatively large mass. Although this combination could provide high sensitivity, it suffers from the large size and temperature and long-term drift due to the unmatched coefficients of thermal expansion (CTE) between the quartz and steel materials, and the bonding material. The development of quartz micromachining techniques makes it possible to fabricate the proof mass structure in a batch process on a thick quartz wafer, and the key feature is to make a flexure thin enough to transfer the inertial force produced by the proof mass to the DETF resonator. Furthermore, the DETF resonator could also be fabricated smaller to provide higher sensitivity to the transferred inertial force. The two-end fixed parts of the DETF should be assembled on the base and proof-mass surface, respectively. To maintain the accelerometer sensitivity and reduce the cross axis sensitivity, the DETF should be precisely mounted onto the base and proof mass structure. From Equation (1), it can be learnt that when a misbonding occurs in the length direction, the applied force would be changed due to the change of force arm. Additionally, when a misbonding occurs in the cross axis, a cross sensitivity will be induced. Unfortunately, it is difficult to align and bond such a miniaturized MEMS resonator in the proper position using traditional gluing methods.

**Figure 2:**
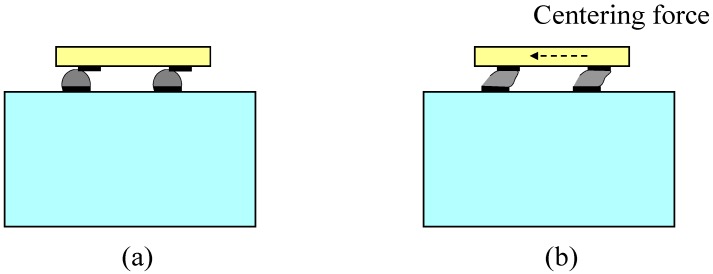
Mechanism of self-alignment: (**a**) misalignment before reflow; (**b**) self-alignment caused by centering force during reflow process.

On the other hand, the flip chip bonding method derived from the IC fabrication technology could mechanically and electrically connect two joint parts. The face to face assembly feature of the process guarantees the shortest connecting wire and a small housing space. Furthermore, the attracting self-alignment function during the reflow process has been thoroughly studied and demonstrated in previous research [12,13,14,15]. Figure 2 shows the self-alignment procedure during the reflow process for flip chip bonding due to the centering force. Recently, flip-chip bonding has been reported for application in packaging MEMS devices [16,17,18], such as barometric pressure sensors [19,20], microspring [21], photonic chips [22], scanner [23], microphones [24] and so on. 

**Figure 3:**
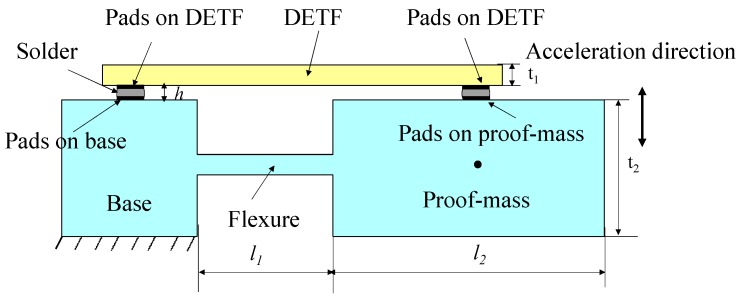
Schematic diagram of the proposed accelerometer.

In this research, the flip-chip bonding method was proposed to realize a DETF based quartz accelerometer by overcoming the mismatch and misalignment problems mentioned above. Figure 3 shows the proposed accelerometer structure. 

The base part would be stabilized and the proof mass would be kept free to allow rotation. The DETF and proof mass structure can be fabricated by the well-established quartz wet etching process on a Z cut wafer with a thickness of 100 μm and 300 μm, respectively [25]. The lead-free gold-tin (AuSn) alloy is selected as the bonding material, because it has excellent mechanical properties with matched CTE with the Z cut quartz wafer [26]. Furthermore, the AuSn alloy is demonstrated to be suitable for fluxless processes, which is very important for MEMS device long term performance [27].

## 2. Design and Fabrication

### 2.1. Structure Design

**Figure 4:**
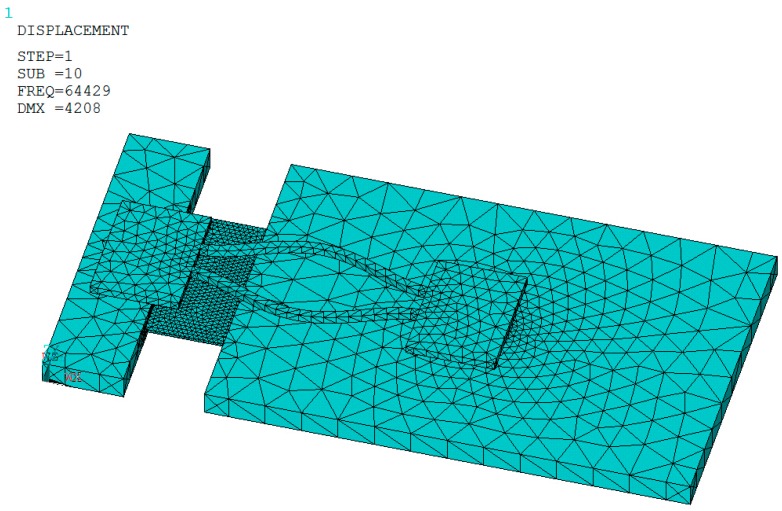
Simulated ninth order in plane anti phase vibration modes.

The full dimensions of the base-proof mass are fixed at 4 mm (width) × 8 mm (length) × 0.3 mm (wafer thickness). The two-end bonding pads are designed at the center of base and proof-mass in the length direction. By considering the final attachment, the base length is fixed at 1 mm. The force applied on the DETF is determined by the following Equation (1):

(1) $$F=Ma\frac{l_{1}+l_{2}}{2}/(\frac{t_{1}+t_{2}}{2}+h)$$

wherein, *l*_1_, *l*_2_ are the flexure, proof-mass length respectively, *l*_1_ + *l*_2_ = 7 mm; *t*_1_, *t*_2_ are the thickness of the DETF, proof-mass, which are 100, 300 μm, respectively; *h* is the height of the bonding solder bump, which is determined by the quantity of solder material and the bonding pad size as shown in Figure 3. M is the mass of proof mass part, which varies on the length (*l*_2_) of proof mass, a is the detecting acceleration which is in the vertical direction. The finite element analysis method (FEM, ANSYS) is used to design and optimize the base-proof, DETF dimensions. The simulation uses free automatic meshing with tetrahedral-shaped elements at the size level of 2. The freedom of the stabilized plane (as shown in Figure 3) is constrained. The DETF full length is determined by the base-proof mass length designed. The DETF resonant frequency is designed to be about 65 KHz by considering the matching oscillation circuit which is usually designed for driving flexural mode vibration below 100 KHz. Figure 4 gives an example picture of established model and simulated vibrating DETF. The desired anti phase flexural vibration is found at the tenth order with a nominal frequency of 64.4 KHz. 

The detailed design of the DETF structure and top and bottom surface excitation electrodes have been reported in other work [28,29]. Generally speaking, small flexure thickness means flexible rotation and high sensitivity. Considering the easy fabrication, the flexure thickness is fixed at 40 μm. To optimize the base-proof mass structure, we just need to concern the maximum force transferred to the DETF as given by Equation (1). A tradeoff should be made between the flexure length *l*_1_ and proof mass length *l*_2_. By fixing the DETF beam dimensions and soldering bump height, and flexure thickness, and considering the complex etching anisotropy of quartz, the proof mass length *l*_2_ is designed to be 6 mm, and accordingly the flexure length *l*_1_ is 1 mm. The width of flexure affects the sensitivity very little and it is fixed at 2 mm by considering the anti-cross axis strength. As expressed in Equation (1), the sensitivity on acceleration decreases with the increase of bump height. Further, by considering the necessary bonding strength, a bump height of 50 μm is preferred. Accordingly, the optimized sizes are 1 mm, 1 mm, 6 mm in length for the base, flexure, and proof mass structure, respectively, 90 μm in width for the DETF beam, and 50 μm in height for the soldering bump, which give an acceleration sensitivity of about 14.5 Hz/g. 

### 2.2. Fabrication

**Figure 5:**
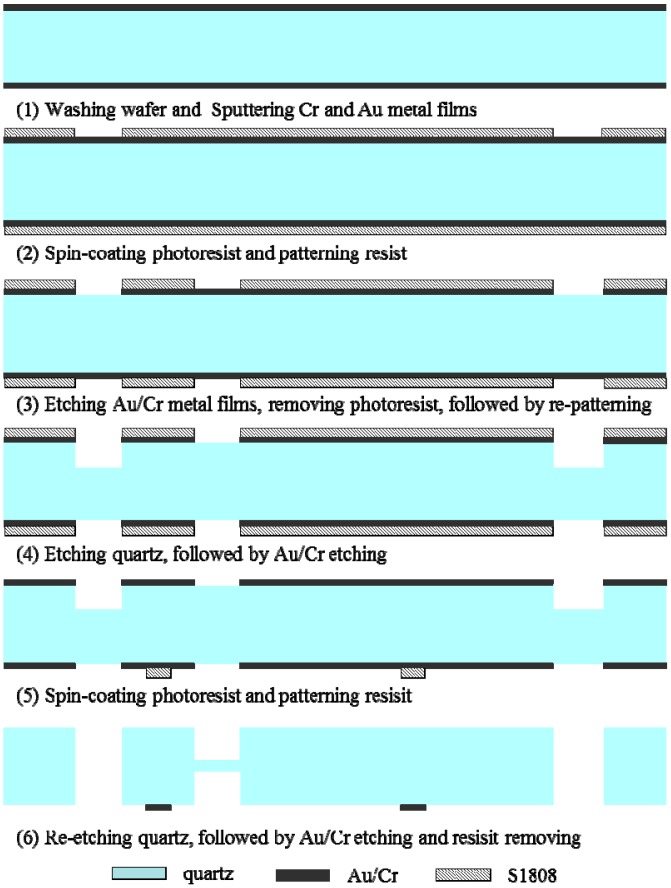
The fabrication process flow of base-proof mass structure.

The DETF fabrication process can also be found in [28,29]. A modified two-step quartz etching process is developed to fabricate the base-proof mass structure with a thinned flexure on a 300 μm thick wafer. Figure 5 shows the fabrication process flow of the base-proof mass structure.

The process is illustrated as follows: (1) Washing quartz wafer using piranha solution (H_2_SO_4_:H_2_O_2_ = 3:1) for 15 min, followed by sputtering chromium (Cr, 30 nm) and gold (Au, 100 nm) bilayer metal films on the double sides. The Au/Cr films are mainly used as quartz etching mask, and some area will be left as bonding pads and leading wire after quartz etching; (2) Spin-coating photoresist on the double surfaces and patterning the top surface. This step defines the structure pattern which will be etched through; (3) Etching the exposed Au/Cr films and removing the photoresist, followed by recoating and patterning photoresist on the double sides. This step is used to define the flexure pattern which will be etched from the double sides and the photoresist should be hard-baked at 150 °C for resisting the aggressive quartz etchant; (4) Etching quartz in saturated ammonium bifluoride solution at 85 °C with a depth of 40 μm, followed by Au/Cr etching and photoresist removal. This step partially etches the quartz and the etched depth should be at least equal to or slightly larger than the designed flexure thickness; (5) Spin-coating and patterning the photoresist. This step defines the bonding pad metal pattern on the bottom surface and (6) Etching quartz, followed by Au/Cr etching and photoresist removal. This step secondly etches the quartz wafer from double sides with a depth of 130 μm achieving the central flexure with a thickness of 40 μm. Finally, the completed wafer is washed with piranha solution to ensure a clean pad surface for the next soldering bonding.

After fabrication, the DETF and base-proof mass structure chips are cut from the respective wafers. The bonding process starts on forming the bump on the base-proof mass pads by reflowing the AuSn21 alloy preform (300 μm × 300 μm × 100 μm) with a peak temperature of 320 °C. After that, the DETF is pre-aligned to the base-proof mass structure using the pad patterns in a face to face manner, followed by another reflow treatment. The bonded sensor chip is assembled into a ceramic package via Ag paste, and the top surface excitation electrodes are wire-bonded onto the base pads to connect with the respective bottom surface excitation electrodes, followed by wire-bonding to the ceramic package pads. The vibration characteristics (Q value, resonant frequency, equivalent circuit parameters) of the fabricated DETF were measured by using impedance analyzer 4294A by contacting the two electrical pads via 42941A probe. Based on the measured equivalent circuit parameters, a multiple common-emitter series oscillator circuit with negative feedback is designed and fabricated to drive the DETF resonator [30].

## 3. Results and Discussion

**Figure 6:**
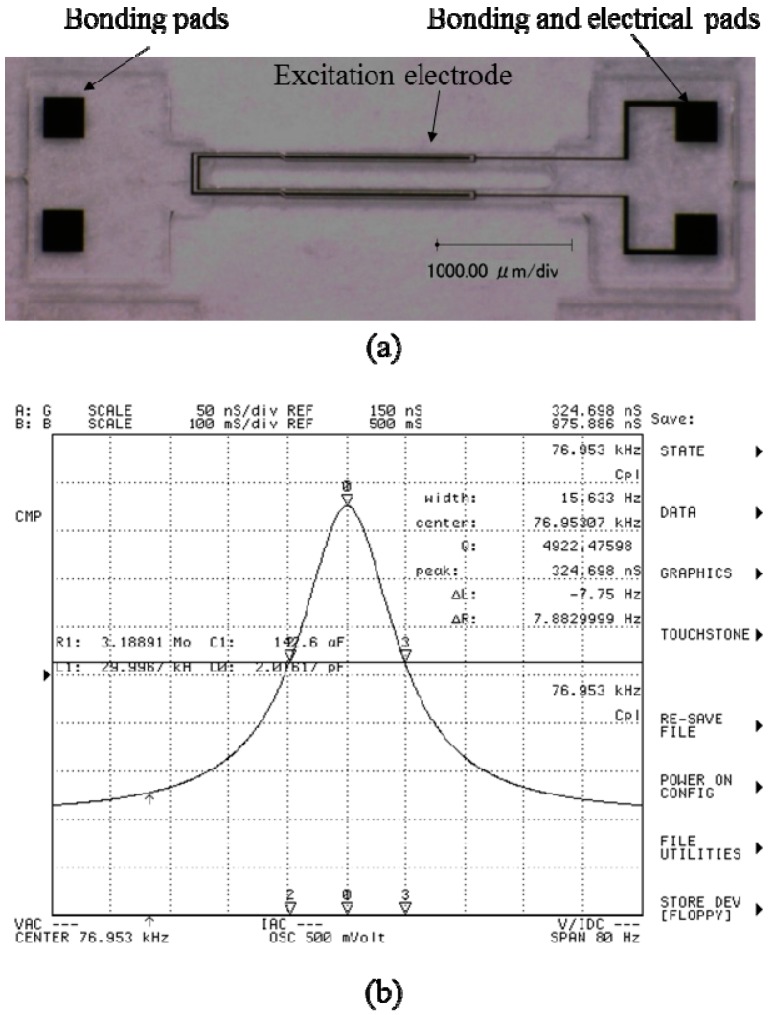
Fabricated DETF: (**a**) Optical view; (**b**) Measured vibration characteristics.

Figure 6a shows an sample optical picture of the fabricated DETF, and Figure 6b shows the conductance characteristics measured using a 4294A impedance analyzer after bonding on the ceramic package. The Q value was measured to be about 5000 and the resonant frequency was 76.9 KHz, which is much higher than the simulated results. The difference between the simulation and measurement is considered to be the cause of the anisotropic etched sidewall along the +X axis [25] which equivalently increases the beam width. 

**Figure 7:**
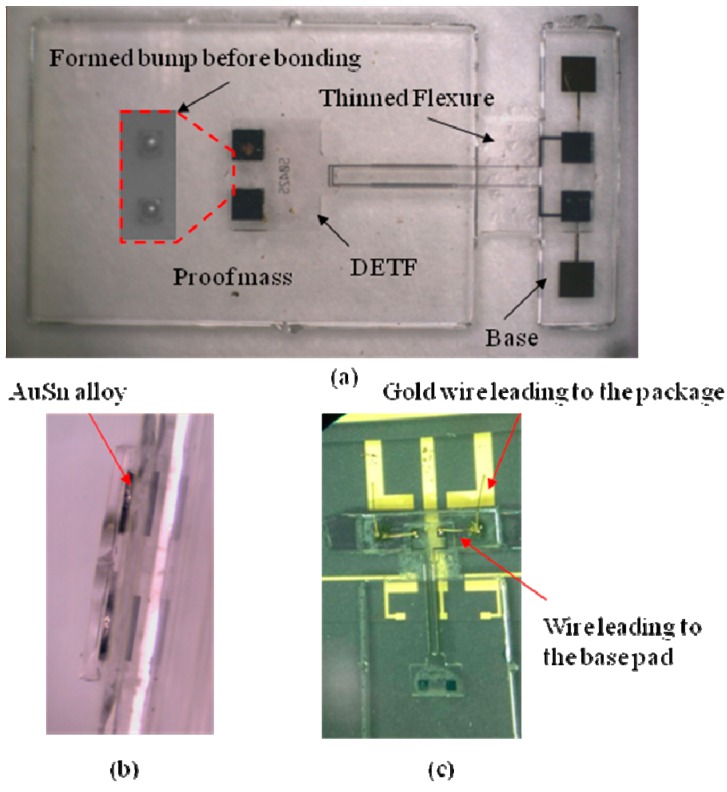
Bonding and packaging of sensor chip: (**a**) Optical picture of base-proof mass structure; (**b**) Side view of the bonding of DETF and base-proof mass structure; (**c**) Close-up picture of the electrodes connection and assembly on the ceramic package

The large motional resistance (3.2 MΩ) is considerable, because this research focuses on demonstrating the flip chip bonding process and only top and bottom surface electrodes are designed. By arranging surrounding electrodes as shown in Figure 1, the motional resistance would be greatly reduced. Figure 7a,b show the bonded DETF and base-proof mass structure and side view of the bonding respectively, and Figure 7c shows the close-up picture of the packaged sensor chip in a ceramic package. Figure 7a demonstrates that the DETF and base-proof mass structure are precisely bonded because the pads on the base and proof mass are completely shadowed by the pads on the DETF. And it can also be seen from the bump shape from the Figure 7b. The bottom and top surface excitation electrodes are electrically led to the base pads via AuSn bump and gold wire, respectively, followed by leading to the ceramic package using gold wire as Figure 7c.

**Figure 8:**
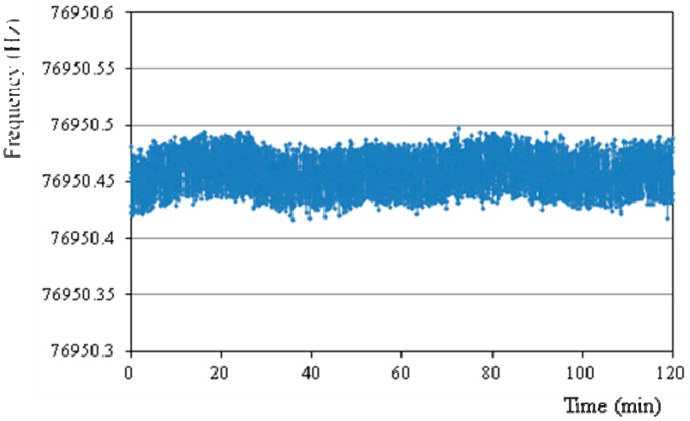
The measured zero bias stability with the oscillation circuit.

Figure 8 shows the sensors bias stability in two hours under room temperature when connected with the fabricated oscillation circuit mentioned above. The frequency output is counted by using an Agilent 53132A frequency meter with a sampling time of 2 s. The calculated standard deviation (1σ) is 0.015 Hz. 

**Figure 9:**
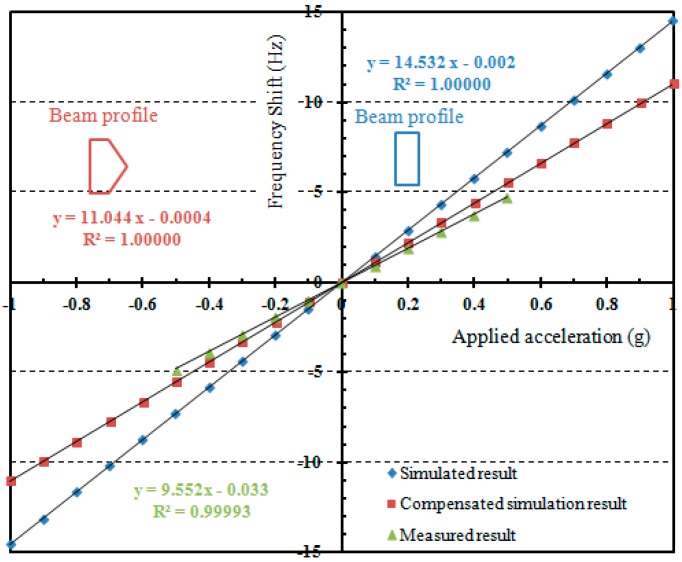
Simulated and measured frequency response under small acceleration.

The sensitivity evaluation is performed by setting the package sensor onto a precise goniometer stage, which has a rotation angle ranging from −30° to 30°. The goniometer is used to generate static gravity acceleration, which corresponds to –0.5 g to 0.5 g. Figure 9 shows the simulated and measured sensitivity results. The measured scale factor is 9.55 Hz/g, which is smaller than the simulated value 14.53 Hz/g. As mentioned above, the existence of large side wall lowers the force-frequency of DETF. By compensating the beam cross section with a side wall as shown in Figure 9 [31] the simulated scale factor becomes 11.04 Hz/g, which is basically consistent with the experimental results. Accordingly, the bias stability corresponds to 1.57 mg. It is considered that there are several main issues to further improve the sensor measurement resolution. The simplest way is to assemble the sensor chip in vacuum, which would greatly increase the Q value and decrease the motional resistance. However, it also means increased cost. The second way is to fabricate the DETF resonator with surrounding excitation electrodes as in Figure 1 to reduce the motional resistance. Finally, a differential sensor structure could also increase the sensor sensitivity and reduce the common mode errors. 

## 4. Conclusions

A digital quartz MEMS accelerometer which includes a force-frequency sensitive DETF resonator and a base-proof mass structure is designed. A simple and effective flip chip bonding method is proposed and demonstrated to be able to precisely align the position of DETF and base-proof mass structure. Utilizing the cheap quartz MEMS wet etching process, the DETF resonator and base-proof mass structure are fabricated and evaluated. The results demonstrate that it can be used as a sensing element. 
